# Physical, Sexual, and Intimate Partner Violence Among Transgender and Gender-Diverse Individuals

**DOI:** 10.1001/jamanetworkopen.2024.19137

**Published:** 2024-06-25

**Authors:** Kalysha Closson, Sabrina C. Boyce, Nicole Johns, David J. Inwards-Breland, Edwin Elizabeth Thomas, Anita Raj

**Affiliations:** 1 Faculty of Health Sciences, Simon Fraser University, Burnaby, British Columbia, Canada; 2 School of Public Health, University of California, Berkeley; 3 Center on Gender Equity and Health, University of California, San Diego; 4 Morehouse School of Medicine, Atlanta, Georgia; 5 Newcomb Institute, Tulane University, New Orleans, Louisiana; 6 Tulane School of Public Health and Tropical Medicine, Tulane University, New Orleans, Louisiana

## Abstract

**Importance:**

Transgender and gender-diverse (TGD) communities experience disproportionate levels of violence, yet due to limitations in measuring TGD identity, few state-representative estimates are available.

**Objective:**

To assess gender identity differences in experiences of violence among adults.

**Design, Setting, and Participants:**

Cross-sectional data from the 2023 California Violence Experiences (CalVEX) survey, weighted to provide state-representative estimates, was used to assess associations between gender identity and past-year experiences of violence among adults 18 years and older. Data were analyzed from June to December 2023.

**Exposure:**

Gender identity (cisgender women, cisgender men, transgender women, transgender men, and nonbinary individuals).

**Main Outcomes and Measures:**

Experience of physical violence (including physical abuse and threat or use of a weapon), sexual violence (verbal sexual harassment, homophobic or transphobic slurs, cyber and physically aggressive sexual harassment, and forced sex), and intimate partner violence (IPV; emotional, physical, or sexual violence) using age-adjusted logistic regression.

**Results:**

In total 3560 individuals (weighted cumulative response rate, 5%) completed the 2023 CalVEX survey, with 1978 cisgender women, 1431 cisgender men, 35 transgender women, 52 transgender men, and 64 nonbinary respondents (mean [SD] age, 47.1 [17.5] years; 635 [17%] were Asian, 839 [37%] were Hispanic, and 1159 [37%] were White). Past-year physical violence was reported by 22 transgender men (43%), 9 transgender women (24%), and 9 nonbinary respondents (14%). Past-year sexual violence was reported by 23 transgender men (42%), 11 transgender women (14%), and 31 nonbinary respondents (56%). Compared with cisgender women, transgender women and transgender men had greater risk of past-year physical violence (any form) (transgender women adjusted incidence rate ratio [AIRR], 6.7; 95% CI, 2.5-18.2; transgender men AIRR, 9.7; 95% CI, 5.3-17.7), as well as past-year IPV (any form) (transgender women AIRR, 3.2; 95% CI, 1.3-8.0; transgender men AIRR, 6.7; 95% CI, 4.0-11.3). Relative to cisgender women, transgender men (AIRR, 3.0; 95% CI, 1.7-5.1) and nonbinary respondents (AIRR, 3.3; 95% CI, 2.1-5.2) had greater risk of past-year sexual violence (any form).

**Conclusions and Relevance:**

In this survey study of adults in California, results showed that TGD individuals, especially transgender men, are at higher risk of experiencing all forms of violence relative to cisgender women. Results highlight the need for gender-affirming violence prevention and intervention services as well as policies that protect TGD individuals from discriminatory violence.

## Key Points

**Question:**

Are transgender and gender-diverse (TGD) individuals at higher risk of experiencing multiple forms of past-year violence?

**Findings:**

Using data from 3560 adults completing a cross-sectional survey, California-representative estimates showed TGD respondents, especially transgender men, were significantly more likely to face physical, sexual, and intimate partner violence in the past year relative to cisgender respondents.

**Meaning:**

These findings suggest there is a critical need for gender-affirming violence prevention, support services, and policies that are protective of TGD individuals.

## Teaser

This survey study evaluates differences in experiences of violence among transgender and gender-diverse individuals compared with cisgender individuals.

## Introduction

Gender-based violence (GBV) is an umbrella term used to document violence directed at a person because of their gender or perceived gender. GBV can include physical, sexual, verbal, and emotional violence in both public and private spaces.^1^ While research on GBV has predominantly focused on cisgender women, there is a growing recognition that GBV also deeply impacts transgender and gender-diverse (TGD) individuals, including transgender men, transgender women, and nonbinary individuals.^2^

The US is seeing a rise in the number of people identifying as TGD due to emerging ways to measure gender identity and increased awareness and acceptance of TGD identities. While estimates of TGD individuals are limited, in the US, it is estimated that over 1.6 million or 0.6% of the adult (older than 18 years) and youth (aged 13-17 years) population identify as transgender.^3^

The growing acknowledgment of the rights and identities of TGD people has brought attention to the elevated risk of violence experienced by TGD individuals. Discrimination, stigma, and systemic challenges create environments in which TGD individuals are disproportionately affected by violence, and there is some indication that increased representation and recognition of TGD people is leading to backlash in terms of both discriminatory policies and violence. Between 2013 and 2019, hate crimes toward TGD individuals increased by 587%.^4^ In the 2015 US Transgender Survey, nearly half of participants (46%) reported verbal harassment and 9% physical violence in the past year for being transgender.^5,6^ These experiences of stigma, discrimination, and violence contribute to elevated gender minority stress,^7^ in which external stressors contribute to long-term physical and mental health consequences.^8,9^

A growing body of research, much of which has been led by transgender and queer communities, underscores the heightened risk for violence faced by TGD individuals,^10^ including that led by the National Center for Transgender Equality and the National Gay and Lesbian Task Force.^11,12,13^ This work has been deeply important in bringing recognition to these neglected populations, but it also has primarily had to rely on nonprobability samples.^10^

At the population level, there remain significant gaps in how to meaningfully enumerate transgender identities and explore experiences of violence across gender identities. Large-scale population health and demographic surveys often fall short in measuring gender comprehensively and/or combine transgender and nonbinary groups to increase statistical power for quantitative analyses.^10^ Consequently, we lack state and national-level data to accurately measure population estimates of different forms of violence for diverse gender groups. Drawing on data from the California Violence Experiences Survey (CalVEX) weighted to provide state-representative estimates, our study seeks to address these gaps by providing a comprehensive analysis of violence experiences across gender identity, including cisgender women and men, transgender women and men, and nonbinary individuals.

## Methods

### Data Source

Data for this study come from CalVEX 2023, a cross-sectional online survey developed by the senior author (A.R.) and conducted with 3560 California adults aged 18 years or older from March 27 to May 28, 2023. The Violence Experiences (VEX) surveys, conducted annually, assess experiences of physical, sexual, and intimate partner violence; discrimination; and related factors such as mental health.^14^

NORC at the University of Chicago implemented CalVEX 2023 with their existing online survey panels, including their nationally representative, probability-based AmeriSpeak panel and supplemented with nonprobability panels from the Lucid nonprobability online opt-in panels to reach the target study sample size (3500 respondents). NORC produces multistage survey weights, such that the weighted study sample was comparable with census estimates of the adult California population with regards to race and ethnicity, education, employment status, age, and disability status. See eMethods in Supplement 1 for details on the sampling procedures, response rate calculations,^15^ and weight construction.

The 2023 survey took approximately 15 minutes to complete, and participants were compensated approximately US $4 for their time. All participants provided consent to NORC for inclusion at the time of panel enrollment. Participants could opt out of the panel and the CalVEX survey entirely or at any time during survey participation. All study procedures were reviewed and approved by institutional review boards at the University of Chicago and University of California San Diego. This study followed the American Association for Public Opinion Research (AAPOR) reporting guidelines.

### Measuring Gender in CalVEX 2023

Our independent variable was gender identity. We designed the survey to use a 2-step approach to capturing gender and transgender identities. We first asked respondents “What is your gender identity?” with response options “Woman; Man; Non-binary/Genderqueer/Gender fluid; Prefer to Self-describe [open text]; Prefer not to answer.” We then separately asked “Do you have lived experience as a trans person?” with answer options “Yes; No; Prefer not to answer.” For this analysis, we created a gender identity variable with 5 categories: men and women reporting experience living as a transgender person were categorized as (1) transgender men or (2) transgender women and those not reporting experience living as a transgender person were categorized as (3) cisgender women and (4) cisgender men. Anyone identifying as nonbinary, genderqueer, genderfluid, or only providing a self-described gender identity not otherwise listed were categorized as (5) nonbinary.

Additionally, among the same participants, gender identity was assessed using the standard NORC gender measure, which asks participants “How do you describe yourself? Male; Female; transgender; or do not identify as male, female, or transgender.” We designed the CalVEX survey to include the additional gender measure because the more simplified NORC one likely excludes nonbinary and transgender individuals in important ways. In addition to not providing a nonbinary response option, the exclusive categorization of a person as either transgender or male or female may lead to misclassification if multiple of those response options is true.^16,17,18,19^

### Past-Year Experiences of Violence

We examined experiences of violence in the past year in several ways. For all forms of violence, participants were first asked whether they had ever experienced that form of violence, and if yes, asked whether they had experienced it in the past year. Detailed definitions and survey items are presented in eTable 1 in Supplement 1.^14^

Physical violence included 3 forms of violence: physical abuse, threat or use of a knife, and threat or use of a gun. Sexual violence included 5 forms of sexual harassment (verbal sexual harassment, transphobic or homophobic sexual harassment, cyber sexual harassment, physically aggressive sexual harassment, and quid pro quo sexual harassment) and sexual assault.

Individuals who experienced physical or sexual violence in the past year were asked where the violence occurred, including public spaces (defined as schools, workplaces, public spaces such as parks and sidewalks, public transit or rideshares, and bars or clubs) and private spaces (defined as a home or a private car). Cyber sexual harassment was characterized as occurring in online and/or virtual spaces.

We separately assessed intimate partner violence, including 22 forms of violence perpetrated by a current or former romantic or sexual partner. It included emotional, controlling, threatening, physical, and sexual forms of violence.

Age, self-reported race and ethnicity, household income, and history of homelessness were assessed; details are included in the eMethods in Supplement 1. Race and ethnicity were assessed to serve as a proxy measure for the social experience of racial and ethnic categories, including experiences of discrimination and marginalization.

### Statistical Analyses

We present descriptive statistics for the population with regards to gender identity and select sociodemographic characteristics. We present prevalence of physical, sexual, and intimate partner violence in the past year by 5 separate gender identities. We conducted pairwise comparisons of prevalences of violence via adjusted Wald test between each gender identity. Cisgender women were used as the reference category because of their known elevated levels of risk for sexual and severe IPV relative to cisgender men.^20,21^ We then conducted Poisson regression models testing the association between gender and past-year experiences of violence, controlling for continuous age in years. Younger age has been shown to be associated with both diverse gender identity^3^ and with greater likelihood of experiencing violence.^14^ In all analyses we applied weights using the *svy* package in Stata version 18.0 (StataCorp) to provide state-representative estimates. Missing data on exposure, outcome, and covariates was 1% or less for all variables, assumed to be missing at random, and complete case analyses were conducted. We evaluated the statistical significance of associations using a threshold of a 2-sided *P* < .05 for all comparisons and 95% CIs for adjusted incidence rate ratios (AIRRs), which are interpreted as risk ratios. Data were analyzed from June to December 2023.

## Results

This analysis included all 3560 respondents to the 2023 CalVEX survey. For the probability sample, 6515 participants were invited to take the survey, and 2144 completed the survey, which yields an AAPOR weighted response rate of 5%. Accounting for survey weighting, the sample was 49% cisgender women (1978 participants), 1% transgender women (35 participants), 48% cisgender men (1431 participants), 1% transgender men (52 participants), and 2% nonbinary individuals (64 participants). The mean (SD) age of all participants was 47.1 (17.5) years; 635 (17%) were Asian, 839 (37%) were Hispanic, and 1159 (37%) were White. Further demographic characteristics of the sample by gender identity are included in Table 1.

**Table 1.** Characteristics of the Sample

Characteristic	Individuals, No. (%)^a^					
	Total (N = 3560)	Cisgender women (n = 1978)	Transgender women (n = 35)	Cisgender men (n = 1431)	Transgender men (n = 52)	Nonbinary (n = 64)
Age, mean (SD), y	47.1 (17.5)	47.3 (18.2)	46.3 (20.5)	47.8 (16.4)	37.9 (16.8)	32.4 (14.0)
Race and ethnicity						
Asian, non-Hispanic	635 (17)	302 (14)	5 (27)	320 (20)	3 (6)	5 (11)
Black, non-Hispanic	730 (5)	452 (5)	5 (2)	257 (6)	9 (6)	7 (1)
Hispanic	839 (37)	455 (37)	11 (39)	322 (35)	23 (57)	28 (59)
White, non-Hispanic	1159 (37)	639 (38)	10 (16)	482 (36)	11 (20)	17 (21)
Multiple or other^b^	197 (4)	130 (5)	4 (16)	50 (3)	6 (11)	7 (8)
History of homelessness (lifetime)						
No	2878 (87)	1621 (88)	18 (44)	1174 (88)	22 (59)	43 (69)
Yes	630 (13)	331 (12)	15 (56)	235 (12)	30 (41)	19 (31)
Household income, US $						
<30 000	896 (22)	569 (24)	10 (19)	271 (18)	19 (29)	27 (57)
30 000 to <60 000	807 (22)	477 (23)	5 (11)	299 (21)	13 (35)	13 (4)
60 000 to <100 000	749 (22)	426 (24)	6 (11)	304 (21)	5 (4)	8 (10)
≥100 000	1108 (35)	506 (29)	14 (60)	557 (40)	15 (31)	16 (29)

^a^ Unweighted number of individuals (survey weighted percentages).

^b^ Other includes participants who selected other, non-Hispanic.

Gender identity as assessed by our 2-item gender and transgender identity measure differed from responses to the NORC single-item measure, which aligns with the US census measure. Categorization based on the 2-item vs single-item measure differed for 4.5% of the survey-weighted sample. Among transgender women, 34 (97%) identified as female in the single item, with the remaining 1 (3%) identifying as transgender. Among transgender men, 43 (84%) identified as male in the single item; 6 (12%) as transgender; 2 (3%) as do not identify as male, female, or transgender; and 1 (1%) as female. Significantly more respondents indicated transgender identity when using the 2-item measure.

Past-year physical violence was reported by 22 transgender men (43%), 9 transgender women (24%), and 9 nonbinary respondents (14%). Past-year physical violence was reported most frequently by transgender men (see Table 2), and was similar across transgender and cisgender women and nonbinary individuals. In a Poisson regression model controlling for age, transgender women (AIRR, 6.7; 95% CI, 2.5-18.2) and transgender men (AIRR, 9.7; 95% CI, 5.3-17.7) had significantly greater risk of past-year physical violence relative to cisgender women (eTable 2 in Supplement 1). Cisgender men (AIRR, 1.5; 95% CI, 0.9-2.4) and nonbinary individuals (AIRR, 2.8; 95% CI, 0.7-10.7) had statistically equivalent risk of past-year physical violence relative to cisgender women.

**Table 2.** Past-Year Physical Violence by Gender Identity and Type

Gender identity	Any physical violence, No. (%)^a^	*P* value^b^	Physical abuse, No. (%)^a^	*P* value^b^	Weapon violence, No. (%)^a^	*P* value^b^
Total	229 (5)	NA	174 (3)	NA	83 (2)	NA
Cisgender women	102 (3)	NA	79 (2)	NA	32 (1)	NA
Transgender women	9 (24)	.10	8 (21)	.11	2 (3)	.58
Cisgender men	87 (5)	.13	61 (3)	.32	39 (2)	.21
Transgender men	22 (43)	<.001	18 (32)	.002	8 (16)	.09
Nonbinary	9 (14)	.28	8 (14)	.27	2 (1)	.71

Abbreviation: NA, not applicable.

^a^ Unweighted number of individuals (survey weighted percentages).

^b^ Wald χ^2^ test *P* value for pairwise comparison with the reference group of cisgender women.

We found that experiences of sexual violence in the past year were reported most frequently by nonbinary individuals (31 participants [56%]) and transgender men (23 participants [42%]) (see Table 3). In a Poisson regression model controlling for age, cisgender men had lower risk (AIRR, 0.5; 95% CI, 0.3-0.7), transgender men had greater risk (AIRR, 3.0; 95% CI, 1.7-5.1), and nonbinary individuals had greater risk (AIRR, 3.3; 95% CI, 2.1-5.2) of past-year sexual violence relative to cisgender women (eTable 3 in Supplement 1). Transgender women (AIRR, 1.4; 95% CI, 0.5-3.5) had statistically equivalent risk of past-year sexual violence relative to cisgender women.

**Table 3.** Past-Year Sexual Violence (SV) by Gender Identity and Type

Gender identity	Any SV, No. (%)^a^	*P* value^b^	Verbal SH, No. (%)^a^	*P* value^b^	HT SH, No. (%)^a^	*P* value^b^	Cyber SH, No. (%)^a^	*P* value^b^	Physically aggressive SH, No. (%)^a^	*P* value^b^	Coercion or quid pro quo SH, No. (%)^a^	*P* value^b^	Forced sex, No. (%)^a^	*P* value^b^
Total	404 (9)	NA	221 (5)	NA	88 (2)	NA	160 (3)	NA	76 (1)	NA	34 (<1)	NA	28 (<1)	NA
Cisgender women	246 (10)	NA	149 (6)	NA	30 (1)	NA	104 (4)	NA	46 (1)	NA	19 (<1)	NA	18 (<1)	NA
Transgender women	11 (14)	.56	6 (4)	.61	4 (5)	.28	4 (4)	.84	5 (5)	.41	1 (<1)	.09	1 (<1)	.38
Cisgender men	93 (5)	<.001	43 (2)	<.001	30 (2)	.40	39 (1)	.004	17 (1)	.52	6 (<1)	.44	5 (<1)	.60
Transgender men	23 (42)	.002	9 (11)	.36	7 (12)	.07	7 (11)	.25	4 (12)	.16	5 (7)	.09	1 (2)	.38
Nonbinary	31 (56)	<.001	14 (39)	.01	17 (26)	.01	6 (3)	.56	4 (1)	.99	3 (1)	.41	3 (<1)	.08

Abbreviations: HT, homophobic and transphobic; NA, not applicable; SH, sexual harassment.

^a^ Unweighted number of individuals (survey weighted percentages).

^b^ Wald χ^2^ test *P* value for pairwise comparison with the reference group of cisgender women.

To further understand experiences of sexual violence, we separated experiences of sexual violence by location—public space, private space, or online and/or virtual space. More than 1 in 3 nonbinary respondents (39%) reported verbal sexual harassment occurring in public spaces. These results are described in the eResults and eTable 4, eTable 5, eTable 6, and eTable 7 in Supplement 1.

We found that experiences of intimate partner violence in the past year were reported most frequently by transgender men (47%), and were elevated for transgender women (18%) and nonbinary individuals (16%) (Table 4). More specifically, we found that 31% of transgender men reported experiencing physical or sexual IPV in the past year. In a Poisson regression model controlling for age, transgender women (AIRR, 3.2; 95% CI, 1.3-8.0) and transgender men (AIRR, 6.7; 95% CI, 4.0-11.3) had greater risk of past-year IPV relative to cisgender women (eTable 8 in Supplement 1). Cisgender men (AIRR, 1.0; 95% CI, 0.6-1.5) and nonbinary individuals (AIRR, 1.9; 95% CI, 0.6-6.4) had statistically equivalent risk of past-year IPV relative to cisgender women.

**Table 4.** Past-Year Intimate Partner Violence (IPV) by Gender Identity and Type

Gender identity	Any IPV, No. (%)^a^	*P* value^b^	Emotional, controlling, or threatening IPV, No. (%)^a^	*P* value^b^	Physical or sexual IPV, No. (%)^a^	*P* value^b^
Total	250 (6)	NA	231 (6)	NA	151 (3)	NA
Cisgender women	119 (5)	NA	110 (5)	NA	76 (3)	NA
Transgender women	10 (18)	.13	10 (18)	.12	8 (12)	.17
Cisgender men	90 (5)	.90	81 (5)	.66	48 (3)	.84
Transgender men	22 (47)	<.001	22 (47)	<.001	12 (31)	.01
Nonbinary	9 (16)	.31	8 (14)	.36	7 (4)	.75

Abbreviation: NA, not applicable.

^a^ Unweighted number of individuals (survey weighted percentages).

^b^ Wald χ^2^ test *P* value for pairwise comparison with the reference group of cisgender women.

## Discussion

In this survey using a sample of California adults weighted to provide state-representative estimates, we found a higher risk of multiple forms of violence particularly for TGD individuals. Compared with cisgender women, who are often centered in GBV research, transgender women and men were more likely to have experienced past-year physical violence, while transgender men and nonbinary individuals were more likely to experience past-year sexual violence. Transgender men faced the greatest risk of IPV in the past year. This study expands upon existing research documenting the elevated prevalence of experiencing violence within TGD communities^2,6,10^ to look at a broad range of violence experiences and documenting differences within TGD subgroups. Our work shows that the association between gender identity and violence was in many instances greatest among transgender men, a demographic group that has often been excluded from GBV research.

Nearly half (47%) of transgender men in our study reported experiencing at least 1 form of IPV in the past year. This was over 40% higher than cisgender women and nearly 30% higher than transgender women. These results are in line with previous research that has documented the critically high levels of IPV facing transgender communities.^10^ Much of the literature on IPV among transgender communities has centered on transgender women. This is likely because much of the transgender and violence research has come from HIV funding, studying populations at disproportionately high risk of acquiring HIV through penile-anal sexual intercourse.^10^ While important, given that transgender men were much more likely to experience IPV in our study, more attention to transgender men and their risk for violence is needed. As such, our results add to the limited body of literature exploring differences in experiences of violence within the transgender community. When we explored these experiences by the type of IPV experienced, transgender men were more likely to report both emotional, controlling, or threatening IPV as well as physical or sexual IPV, with 31% of transgender men reporting experiencing physical or sexual IPV in the past year, compared with only 3% of the total sample. Given these stark numbers, it is essential that IPV screening and services are expanded to ensure they are accessible and safe for transgender men. Current IPV services are often framed as violence against women services and thus may not feel welcoming or accessible for transgender men.^22,23^

Nonbinary individuals also faced elevated risk of violence in the past year when compared with cisgender women. Over half of nonbinary individuals reported experiencing some form of sexual violence in the past year, with verbal sexual harassment being the most common form of sexual violence reported. More than 1 in 3 nonbinary respondents (39%) reported verbal sexual harassment occurring in public spaces. Critically high levels of harassment experienced by nonbinary respondents are in line with national reports and research documenting experiences of harassment by TGD individuals in public spaces such as workplaces and public bathrooms.^24,25^ In light of the growing anti-LGBTQ+ movements across the US and the globe and reductions in legal protections for lesbian, gay, bisexual, transgender, queer, and other individuals (LGBTQ+), there are concerns that experiences of violence and harassment toward LGBTQ+ communities are being legitimized and will continue to grow.^26^ As such, efforts to enact laws and policies that protect TGD communities from violence and discrimination are critically needed. Moreover, support services and law enforcement will require concerted effort to build trust with transgender and nonbinary communities in order for TGD communities to be willing to report experiences and seek protections given historical and current harm by these institutions.^27^

With the additional measure of gender identity in the 2023 CalVEX survey, we were able to more accurately categorize a greater number of TGD individuals than seen in prior rounds of this survey, conducted annually since 2020. In asking about gender identity and experience living as a transgender person, we acknowledge that transgender women are women and transgender men are men and that nonbinary, gender queer, and gender fluid are important gender categories that should explicitly be asked about in population-based surveys.^17^ We acknowledge that gender identity discourses are continuously in flux and that continual efforts are needed to engage with TGD communities to ensure that the ways in which we ask and present on gender identity safely represent TGD communities and works to prevent harm in the process.^28^

### Limitations

This study had limitations. The primary limitation of this study is the relatively small samples of transgender and nonbinary respondents, which can affect observance of significant and stable findings and limit our ability to conduct within-group analyses that are important for understanding intersectional experiences of gender identity and violence. For example, transgender and nonbinary individuals who belong to minoritized racial and ethnic groups face disproportionate risk of violence and are at greatest risk of fatal violence,^24^ but we could not assess this in our study due to small cell sizes. Exact point estimates presented here should be interpreted with caution, although the striking differences in risk estimates between gender identities even with such limited samples are notable. Both within- and between-gender identity diversity should be further explored within larger samples. Additionally, the sampling design includes a nonprobability sample to supplement the AmeriSpeak probability sample. While we have systematically implemented validated multistage weighting procedures to offset sampling and nonresponse bias, remaining bias in these estimates likely still exists, especially for subsamples (like TGD respondents) that are particularly small in the unweighted sample, limiting the state-representativeness of findings on such subpopulations. This study, however, is the only effort known to date to provide state-representative estimates for a comprehensive range of violence experiences among adults in California and relevant subpopulations, so offers a rigorous attempt at furthering our understanding of violence prevalences. We also note that our study is limited in capturing representation from houseless individuals due to the household-based sampling method, which may be a particular limitation for TGD communities, given that 56% of transgender women, 41% of transgender men, and 31% of nonbinary respondents reported a history of homelessness in our study.

## Conclusions

Findings highlight that relative to cisgender women, TGD indviduals experienced disproportionate levels of violence in the past year. These estimates often go unnoticed in large-scale population-based surveys given limitations and gaps in measuring gender identity. An updated measure of gender identity in our 2023 survey allowed for greater identification of TGD individuals. This approach may guide future population-based surveys in their measurement of gender identity to support greater engagement and more accurate enumeration of TGD communities. Results further revealed that transgender men faced the greatest levels of past-year violence, with especially high levels of IPV. Overall findings highlight the need to enhance and expand gender-affirming violence prevention and intervention services, as well as policies that protect TGD individuals from further violence and discrimination.

## Notes

Supplement 1.
eMethods.

eResults.
**eTable 1.** Definitions of Violence Provided in Survey **eTable 2.** Unadjusted and Adjusted Poisson Regression Analyses Examining Association Between Gender Identity and Past-Year Physical Violence Experience, Any Form **eTable 3.** Unadjusted and Adjusted Poisson Regression Analyses Examining Association Between Gender Identity and Past-Year Sexual Violence Experience, Any Form **eTable 4. **Past-Year Sexual Violence (SV) in Public Spaces, Overall and by Type **eTable 5.** Past-Year Sexual Violence in Private Spaces, Overall and by Type **eTable 6. **Past-Year Cyber Sexual Harassment **eTable 7.** Unadjusted and Adjusted Poisson Regression Analyses Examining Association Between Gender Identity and Past-Year Sexual Experience, Any Form, in Public and Private Spaces **eTable 8.** Unadjusted and Adjusted Poisson Regression Analyses Examining Association Between Gender Identity and Past-Year Intimate Partner Violence Experience, Any Form

Supplement 2.
Data Sharing Statement
