# Supplementary material for: Physical, Sexual, and Intimate Partner Violence Among Transgender and Gender-Diverse Individuals
Source: JAMA Netw Open. 2024 Jun 25;7(6):e2419137. doi: 10.1001/jamanetworkopen.2024.19137 (PMC11200137; doi:10.1001/jamanetworkopen.2024.19137)
Supplement: Supplement 1. — eMethods. eResults. eTable 1. Definitions of Violence Provided in Survey eTable 2. Unadjusted and Adjusted Poisson Regression Analyses Examining Association Between Gender Identity and Past-Year Physical Violence Experience, Any Form eTable 3. Unadjusted and Adjusted Poisson Regression Analyses Examining Association Between Gender Identity and Past-Year Sexual Violence Experience, Any Form eTable 4. Past-Year Sexual Violence (SV) in Public Spaces, Overall and by Type eTable 5. Past-Year Sexual Violence in Private Spaces, Overall and by Type eTable 6. Past-Year Cyber Sexual Harassment eTable 7. Unadjusted and Adjusted Poisson Regression Analyses Examining Association Between Gender Identity and Past-Year Sexual Experience, Any Form, in Public and Private Spaces eTable 8. Unadjusted and Adjusted Poisson Regression Analyses Examining Association Between Gender Identity and Past-Year Intimate Partner Violence Experience, Any Form [file jamanetwopen-e2419137-s001.pdf]

# Supplemental Online Content

Closson K, Boyce SC, Johns N, Inwards-Breland DJ, Thomas EE, Raj A. Physical, sexual, and intimate partner violence among transgender and gender diverse individuals. *JAMA Netw Open*. 2024;7(6):e2419137. doi:10.1001/jamanetworkopen.2024.19137

**eMethods.**
**eResults.**
**eTable 1.** Definitions of Violence Provided in Survey
**eTable 2.** Unadjusted and Adjusted Poisson Regression Analyses Examining Association Between Gender Identity and Past-Year Physical Violence Experience, Any Form
**eTable 3.** Unadjusted and Adjusted Poisson Regression Analyses Examining Association Between Gender Identity and Past-Year Sexual Violence Experience, Any Form
**eTable 4.** Past-Year Sexual Violence (SV) in Public Spaces, Overall and by Type
**eTable 5.** Past-Year Sexual Violence in Private Spaces, Overall and by Type
**eTable 6.** Past-Year Cyber Sexual Harassment
**eTable 7.** Unadjusted and Adjusted Poisson Regression Analyses Examining Association Between Gender Identity and Past-Year Sexual Experience, Any Form, in Public and Private Spaces
**eTable 8.** Unadjusted and Adjusted Poisson Regression Analyses Examining Association Between Gender Identity and Past-Year Intimate Partner Violence Experience, Any Form

This supplemental material has been provided by the authors to give readers additional information about their work.

© 2024 Closson K et al. *JAMA Network Open.*

## eMethods

We followed the American Association for Public Opinion Research (AAPOR) reporting guidelines for survey research in developing this manuscript.

### Sampling and response rates

The AmeriSpeak panel, which makes up 60% of this study's sample, uses a multi-stage area probability and address-based sampling approach to recruit participants, with a known, non-zero probability of selection from the NORC National Sample Frame. The American Association for Public Opinion Research Response Rate calculation 3 (AAPOR RR3), which is a response rate methodology to standardize such calculations across studies, particularly online surveys, was used to calculate the response rate.[1] The response rate is calculated as I/((I+P)+(R+NC+O)+e(UH+UO)), where I indicates complete responses, P partial responses, R refusals, NC non-contact, O other, UH unknown household, UO unknown other. This approach, different from crude response rate calculations, includes in the denominator e, an estimated proportion of cases of unknown eligibility that are eligible, to further minimize unknown non-response bias. The non-probability sample, which makes up the remainder of the study sample, include respondents from the Lucid nonprobability online opt-in panels. Because the sampling frame is unknown for a non-probability sample, a response rate is not calculated.

### Survey weights

NORC produces multi-stage survey weights in three stages. First, probability and non-probability sample weights are developed separately. For the probability sample, NORC calculates panel weights, adjusts for sample selection within the panel for the study, adjusts for non-response, trims extreme responses, and calibrates the probability weight to benchmarks through raking ratio adjustment. For the non-probability sample, baseweights are set to one for all cases and then calibrated to population benchmarks through raking ratio adjustment. Next, statistical corrections were made to the weights to incorporate the non-probability sample using NORC's TrueNorth™ calibration services, an approach that uses a small area model to support domain-level estimates, where the domains were defined by race/ethnicity, age, education, and California region, and included external covariates from the American Community Survey. The study design effect was 2.39, with a study margin of error of +/-2.73%, a measure of uncertainty that accounts for the variability associated with the probability sample as well as the potential bias associated with the nonprobability sample. The weighted sample may slightly under-represent foreign-born and non-citizen Californians and may have slightly lower than state median household income; however, these differences from official state figures are not substantial.

### Measurement of sociodemographic characteristics

Age, race/ethnicity, household income, and history of homelessness were assessed. Age was measured continuously. Race/ethnicity was assessed as a proxy for structural and societal racism using a measure selected by NORC for the AmeriSpeak panel. Respondents were categorized based on their selection of the following options (check all that apply): Non-Hispanic Asian/Pacific Islander, Black, White, Other; if more than one Non-Hispanic race was selected, respondents were classified as "Two or more races." Participants who, in the same question, selected Hispanic were categorized as such. NORC categorized responses in the dataset received by the research team. Household income was categorized as <$30,000, $30,000-59,999, $60,000-$99,999, and $100,000 or more. History of homelessness was measured with the single-item question, "Have you ever been homeless – on the streets or in a shelter?" with response options of "yes, in the past year", "yes, but not in the past year", and "never"; any "yes" response was categorized as having had a history of homelessness.

© 2024 Closson K et al. *JAMA Network Open.*

# eResults

**Results on sexual violence experiences in public and private spaces (Supplemental eTable 7)**

To further understand experiences of sexual violence, we separated experiences of sexual violence by location – public space, private space, or online/virtual space. Sexual violence in public spaces was reported by 46% of nonbinary respondents, 28% of transgender men, 14% of transgender women, and 7% of cisgender women. Sexual violence in private spaces was reported by 28% of nonbinary respondents, 24% of transgender men, 3% of transgender women, and 2% of cisgender women. Transgender men reported the highest levels of online sexual harassment (11%) with lower prevalences reported by all other groups (1-4%). Nonbinary individuals and transgender men were more likely than cisgender women to experience sexual violence in both public and private spaces; no significant difference was observed for online/virtual spaces (see Supplemental eTables 4-6).

In a Poisson regression model controlling for age, cisgender men had lower risk [AIRR 0.5, 95% CI 0.3-0.8], transgender men had greater risk [AIRR 2.7, 95% CI 1.3-5.4], and nonbinary respondents had greater risk [AIRR 3.7, 95% CI 2.3-5.9] of past year sexual violence in public spaces relative to cisgender women (see Supplemental eTable 7). Transgender women [AIRR 2.0, 95% CI 0.7-5.4] had statistically equivalent risk of past year sexual violence in public spaces relative to cisgender women. Additionally, transgender men [AIRR 11.8, 95% CI 5.0-27.5] and nonbinary respondents [AIRR 11.8, 95% CI 4.4-31.5] had greater risk of past year sexual violence in private spaces relative to cisgender women. Transgender women [AIRR 2.1, 95% CI 0.4-10.5] and cisgender men [AIRR 1.0, 95% CI 0.5-2.1] had statistically equivalent risk of past year sexual violence in private spaces relative to cisgender women. Due to small numbers of reported experiences in private spaces, these estimates should be considered with caution.

© 2024 Closson K et al. *JAMA Network Open.*

## eTable 1. Definitions of Violence Provided in Survey.

| *Physical and sexual violence definitions* | |
|---|---|
| **Physical violence** | ***Prompt:*** *Please check off each type of experience that has ever been done to you.*<br><br>1. Physical abuse including being hit, slapped, punched, shoved, choked, kicked, shaken or otherwise physically hurt.<br>2. Being threatened or hurt with a knife<br>3. Being threatened or hurt with a gun. |
| **Sexual violence** | A summary variable including verbal sexual harassment, homophobic/transphobic comments, cyber sexual harassment, physically aggressive sexual harassment, quid pro quo sexual harassment or coercion, and forced sex.<br><br>***Prompt****: This set of questions asks you about sexually harassing or abusive experiences you may have had in your life. Please check off each type of experience that have ever been done to you.*<br>1. Verbal sexual harassment<br>2. Homophobic or transphobic comments<br>3. Cyber sexual harassment<br>4. Physically aggressive sexual harassment<br>5. Quid pro quo sexual harassment or coercion<br>6. Forced sex<br><br>(See definitions below provided to survey respondents for each form of sexual violence.) |
| **Verbal sexual harassment** | Defined as someone whistling, leering or staring at you, or calling out to you in ways that make you feel disrespected or unsafe. It can include someone talking about your body parts (such as your butt or breasts) inappropriately or offensively or saying sexually explicit comments or questions ("I want to do BLANK to you"). It can also include someone repeatedly asking you for a date or your phone number when you've said no. |
| **Homophobic or transphobic comments** | Defined as someone misgendering you or calling you a homophobic or transphobic slur, like "Fag," "Dyke," or "Tranny." |
| **Cyber sexual harassment** | Defined on someone electronically sending you or showing you sexual content without your permission, such as over e-mail, social media, or on their phone or computer. This includes someone taking and/or sharing sexual pictures or videos of you without your permission. |
| **Physically aggressive sexual harassment** | Defined as someone flashing or exposing their genitals to you without your permission, someone purposely touching you or brushing up against you in an unwelcome, sexual way. |

© 2024 Closson K et al. *JAMA Network Open.*

| *Physical and sexual violence definitions* | |
|---|---|
| **Quid pro quo sexual harassment or coercion** | Defined as someone forcing or pressuring you to do a sexual act in exchange for something (such as a good grade, a promotion, a job, drugs, food, money, or something similar) or instead of something (like paying rent or a citation, etc.). |
| **Forced sex** | Defined as someone forcing you to do a sexual act without your permission or one that you don't want to do (including while you are under the influence of alcohol or drugs). |
| **Intimate partner violence** | This was measured with the following question and response options, adapted from items in the Composite Abuse Scale (Revised)-Short Form (CASR-SF)[2] and from the National Intimate Partner and Sexual Violence Survey (NISVS) study conducted by the Centers for Disease Control and Prevention[3]:<br><br>***Prompt:*** *The following set of questions asks you about difficulties or mistreatment from a current or former romantic or sexual partner. This can include a spouse, someone you were casually dating, boyfriends, girlfriends, or sexual partners.*<br><br>Has a current or ex- romantic or sexual partner ever done any of the following when you did not want them to?<br>1. Insulted, humiliated, or made fun of you in front of others<br>2. Kept you from having your own money<br>3. Tried to keep you from seeing or talking to your family or friends<br>4. Kept track of you by demanding to know where you were and what you were doing<br>5. Made threats to physically harm you<br>6. Made threats to harm someone close to you<br>7. Threatened to hurt themselves or commit suicide because they were upset with you<br>8. Made decisions for you that should have been yours to make<br>9. Destroyed something that was important to you<br>10. Intentionally hurt or threatened to use violence against your pet(s)<br>11. Harassed you by phone, text, email or using social media<br>12. Slapped you<br>13. Pushed or shoved you<br>14. Hit you with a fist or something hard<br>15. Hurt you by pulling your hair<br>16. Slammed you against something<br>17. Tried to hurt you by choking or suffocating you<br>18. Beaten you<br>19. Burned you on purpose<br>20. Used a knife on you<br>21. Used a gun on you<br>22. Forced or tried to force you to have sex, or made you perform sexual acts that you did not want to perform |

## eTable 2. Unadjusted and Adjusted Poisson Regression Analyses Examining Association Between Gender Identity and Past-Year Physical Violence Experience, Any Form

| | Unadjusted | | | Adjusted[a] | | |
|---|---|---|---|---|---|---|
| | **IRR** | **95% CI** | **p-value** | **AIRR** | **95% CI** | **p-value** |
| **Cisgender women** | *Ref* | *Ref* | *Ref* | *Ref* | *Ref* | *Ref* |
| **Transgender women** | 6.9 | 2.4,20.1 | <0.001 | 6.7 | 2.5,18.2 | <0.001 |
| **Cisgender men** | 1.5 | 0.9,2.4 | 0.13 | 1.5 | 0.9,2.4 | 0.12 |
| **Transgender men** | 12.5 | 6.8,23.0 | <0.001 | 9.7 | 5.3,17.7 | <0.001 |
| **Nonbinary** | 4.2 | 1.0,17.3 | 0.048 | 2.8 | 0.7,10.7 | 0.13 |

[a]Adjusted for respondent age, continuous

## eTable 3. Unadjusted and Adjusted Poisson Regression Analyses Examining Association Between Gender Identity and Past-Year Sexual Violence Experience, Any Form

| | Unadjusted | | | Adjusted[a] | | |
|---|---|---|---|---|---|---|
| | **IRR** | **95% CI** | **p-value** | **AIRR** | **95% CI** | **p-value** |
| **Cisgender women** | *Ref* | *Ref* | *Ref* | *Ref* | *Ref* | *Ref* |
| **Transgender women** | 1.4 | 0.5,3.6 | 0.50 | 1.4 | 0.5,3.5 | 0.53 |
| **Cisgender men** | 0.5 | 0.3,0.7 | <0.001 | 0.5 | 0.3,0.7 | <0.001 |
| **Transgender men** | 4.0 | 2.4,6.8 | <0.001 | 3.0 | 1.7,5.1 | <0.001 |
| **Nonbinary** | 5.4 | 3.5,8.3 | <0.001 | 3.3 | 2.1,5.2 | <0.001 |

[a]Adjusted for respondent age, continuous

© 2024 Closson K et al. *JAMA Network Open.*

**eTable 4. Past-Year Sexual Violence (SV) in Public Spaces, Overall and by Type [Includes School, Workplace, Public Space, Public Transit/Rideshare, Bar/Club]**

| | Any SV in public space | p-value[b] | Verbal SH | p-value[b] | Homo/Trans phobic SH | p-value[b] | Physically aggressive SH | p-value[b] | Coercion/Quid pro quo SH | p-value[b] | Forced sex | p-value[b] |
|---|---|---|---|---|---|---|---|---|---|---|---|---|
| | n (%)[a] | | n (%)[a] | | n (%)[a] | | n (%)[a] | | n (%)[a] | | n (%)[a] | |
| ***Total*** | *264 (6%)* | | *179 (4%)* | | *73 (2%)* | | *50 (1%)* | | *21 (0%)* | | *9 (0%)* | |
| **Cisgender women** | 153 (7%) | ref | 121 (5%) | ref | 22 (1%) | ref | 27 (1%) | ref | 7 (0%) | ref | 6 (0%) | ref |
| **Transgender women** | 10 (14%) | 0.29 | 5 (4%) | 0.72 | 4 (5%) | 0.26 | 4 (5%) | 0.36 | 1 (0%) | 0.19 | 0 (0%) | 0.22 |
| **Cisgender men** | 59 (4%) | 0.01 | 33 (2%) | <0.001 | 26 (3%) | 0.55 | 13 (1%) | 0.85 | 6 (1%) | 0.09 | 1 (0%) | 0.62 |
| **Transgender men** | 17 (28%) | 0.01 | 7 (9%) | 0.40 | 7 (12%) | 0.07 | 3 (5%) | 0.26 | 5 (7%) | 0.08 | 1 (2%) | 0.34 |
| **Nonbinary** | 25 (46%) | 0.001 | 13 (39%) | 0.004 | 14 (12%) | 0.04 | 3 (1%) | 0.73 | 2 (1%) | 0.34 | 1 (0%) | 0.25 |

[a]Unweighted Ns (Survey weighted percentages)
[b]Wald Chi-Squared Test p-value for pairwise comparison with the reference group of cisgender women
Abbreviations: sexual violence, SV; sexual harassment, SH

© 2024 Closson K et al. *JAMA Network Open.*

## eTable 5. Past-Year Sexual Violence in Private Spaces, Overall and by Type [Includes Home or Private Car]

| | Any SV in private space | p-value[b] | Verbal SH | p-value[b] | Homo/Trans phobic SH | p-value[b] | Physically aggressive SH | p-value[b] | Coercion/Quid pro quo SH | p-value[b] | Forced sex | p-value[b] |
|---|---|---|---|---|---|---|---|---|---|---|---|---|
| | n (%)[a] | | n (%)[a] | | n (%)[a] | | n (%)[a] | | n (%)[a] | | n (%)[a] | |
| ***Total*** | *129 (2%)* | | *77 (1%)* | | *32 (1%)* | | *33 (1%)* | | *22 (0%)* | | *22 (0%)* | |
| **Cisgender women** | 71 (2%) | ref | 47 (1%) | ref | 12 (1%) | ref | 17 (0%) | ref | 13 (0%) | ref | 14 (0%) | ref |
| **Transgender women** | 4 (3%) | 0.50 | 3 (3%) | 0.49 | 1 (1%) | 0.77 | 2 (0%) | 0.24 | 1 (0%) | 0.18 | 1 (0%) | 0.72 |
| **Cisgender men** | 29 (2%) | 0.95 | 16 (1%) | 0.79 | 6 (0%) | 0.34 | 11 (0%) | 0.63 | 3 (0%) | 0.70 | 4 (0%) | 0.67 |
| **Transgender men** | 11 (24%) | 0.01 | 6 (6%) | 0.10 | 4 (11%) | 0.09 | 2 (9%) | 0.20 | 3 (4%) | 0.19 | 1 (2%) | 0.36 |
| **Nonbinary** | 14 (28%) | 0.01 | 5 (12%) | 0.23 | 9 (18%) | 0.04 | 1 (0%) | 0.17 | 2 (0%) | 0.50 | 2 (0%) | 0.20 |

[a]Unweighted Ns (Survey weighted percentages)
[b]Wald Chi-Squared Test p-value for pairwise comparison with the reference group of cisgender women
Abbreviations: sexual violence, SV; sexual harassment, SH

## eTable 6. Past-Year Cyber Sexual Harassment [SH; Online/Virtual]

| | Cyber sexual harassment | p-value[b] |
|---|---|---|
| | *n (%)[a]* | |
| ***Total*** | *160 (3%)* | |
| **Cisgender women** | 104 (4%) | ref |
| **Transgender women** | 4 (4%) | 0.84 |
| **Cisgender men** | 39 (1%) | 0.004 |
| **Transgender men** | 7 (11%) | 0.25 |
| **Nonbinary** | 6 (3%) | 0.56 |

[a]Unweighted Ns (Survey weighted percentages)
[b]Wald Chi-Squared Test p-value for pairwise comparison with the reference group of cisgender women

© 2024 Closson K et al. *JAMA Network Open.*

**eTable 7. Unadjusted and Adjusted Poisson Regression Analyses Examining Association Between Gender Identity and Past-Year Sexual Experience, Any Form, in Public and Private Spaces**

| | Public spaces | | | | | | Private spaces | | | | | |
|---|---|---|---|---|---|---|---|---|---|---|---|---|
| | Unadjusted | | | Adjusted | | | Unadjusted | | | Adjusted | | |
| | IRR | 95% CI | p-value | AIRR | 95% CI | p-value | IRR | 95% CI | p-value | AIRR | 95% CI | p-value |
| **Cisgender women** | *Ref* | *Ref* | *Ref* | *Ref* | *Ref* | *Ref* | *Ref* | *Ref* | *Ref* | *Ref* | *Ref* | *Ref* |
| **Transgender women** | 2.1 | 0.8,5.5 | 0.15 | 2.0 | 0.7,5.4 | 0.17 | 2.1 | 0.4,10.8 | 0.35 | 2.1 | 0.4,10.5 | 0.37 |
| **Cisgender men** | 0.5 | 0.3,0.8 | 0.01 | 0.5 | 0.3,0.8 | 0.01 | 1.0 | 0.5,2.1 | 0.95 | 1.0 | 0.5,2.1 | 0.93 |
| **Transgender men** | 4.0 | 2.1,7.6 | <0.001 | 2.7 | 1.4,5.4 | 0.004 | 15.3 | 6.5,35.6 | <0.001 | 11.8 | 5.0,27.5 | <0.001 |
| **Nonbinary** | 6.7 | 3.9,11.5 | <0.001 | 3.7 | 2.3,5.9 | <0.001 | 17.8 | 7.4,43.0 | <0.001 | 11.8 | 4.4,31.5 | <0.001 |

[a]Adjusted for respondent age, continuous

**eTable 8. Unadjusted and Adjusted Poisson Regression Analyses Examining Association Between Gender Identity and Past-Year Intimate Partner Violence Experience, Any Form**

| | Unadjusted | | | Adjusted[a] | | |
|---|---|---|---|---|---|---|
| | IRR | 95% CI | p-value | AIRR | 95% CI | p-value |
| **Cisgender women** | *Ref* | *Ref* | *Ref* | *Ref* | *Ref* | *Ref* |
| **Transgender women** | 3.3 | 1.3,8.6 | 0.02 | 3.2 | 1.3,8.0 | 0.01 |
| **Cisgender men** | 1.0 | 0.6,1.5 | 0.90 | 1.0 | 0.6,1.5 | 0.91 |
| **Transgender men** | 8.8 | 5.1,15.2 | <0.001 | 6.7 | 4.0,11.3 | <0.001 |
| **Nonbinary** | 2.9 | 0.8,10.7 | 0.11 | 1.9 | 0.6,6.4 | 0.30 |

[a]Adjusted for respondent age, continuous

© 2024 Closson K et al. *JAMA Network Open.*

## eReferences

1. The American Association for Public Opinion Research (2016). “Standard Definitions: Final Dispositions of Case Codes and Outcome Rates for Surveys.” Accessed January 16, 2024: https://www.aapor.org/Standards-Ethics/Standard-Definitions-(1).aspx.
2. Ford-Gilboe, M., Wathen, C. N., Varcoe, C., MacMillan, H. L., Scott-Storey, K., Mantler, T., Hegarty, K., & Perrin, N. (2016). Development of a brief measure of intimate partner violence experiences: the Composite Abuse Scale (Revised)-Short Form (CASR-SF). BMJ open, 6(12), e012824. https://doi.org/10.1136/bmjopen-2016-012824
3. Kresnow M, Smith SG, Basile KC, Chen J. The National Intimate Partner and Sexual Violence Survey: 2016/2017 methodology report. Atlanta, GA: Centers for Disease Control and Prevention, National Center for Injury Prevention and Control; 2021.

© 2024 Closson K et al. *JAMA Network Open.*
