# Supplementary material for: Physical, Sexual, and Intimate Partner Violence Among Transgender and Gender-Diverse Individuals
Source: JAMA Netw Open. 2024 Jun 25;7(6):e2419137. doi: 10.1001/jamanetworkopen.2024.19137 (PMC11200137; doi:10.1001/jamanetworkopen.2024.19137)
Supplement: Supplement 2. — Data Sharing Statement [file jamanetwopen-e2419137-s002.pdf]

Data Sharing Statement

Closson. Physical, Sexual, and Intimate Partner Violence Among Transgender and Gender-Diverse Individuals. *JAMA Netw Open*. Published June 25, 2024.
doi:10.1001/jamanetworkopen.2024.19137

Data
Data available: Yes
Data types: Deidentified participant data
How to access data: Data are currently publicly available here:
https://www.openicpsr.org/openicpsr/project/199087/version/V1/view.
When available: With publication

Supporting Documents
Document types: Statistical/analytic code
How to access documents: Analytic code is available upon request by emailing
nejohns@health.ucsd.edu.
When available: beginning date: 04-01-2024

Additional Information
Who can access the data: Anyone
Types of analyses: Any purpose
Mechanisms of data availability: accessing data from website
